# Supplementary material for: Graph-enhanced deep learning for diabetic retinopathy diagnosis: A quality-aware and uncertainty-driven approach
Source: PLoS Comput Biol. 2025 Dec 5;21(12):e1013745. doi: 10.1371/journal.pcbi.1013745 (PMC12680365; doi:10.1371/journal.pcbi.1013745)
Supplement: Attachment — Submitted filename: PCOMPBIOL-D-25-00649_Response.pdf [file pcbi.1013745.s001.pdf]

Manuscript Number: PCOMPBIOL-D-25-00649

Reply to Reviewers

Graph-Enhanced Deep Learning for Diabetic Retinopathy Diagnosis: A Quality-Aware and Uncertainty-Driven Approach

We would like to thank the reviewers for their comments. In the following, we will present our responses to the comments together with a summary of the corresponding changes in the revised manuscript in Yellow Highlights. All page numbers mentioned below are referred to in the new revised manuscript.

Reviewer 1

General Comment: Thank you for allowing me to review this work. I have gone through it and have the following comments:

Comment 1. Add the fullform of CNN and DR in the abstract.
Reply: We have included the full forms—"Diabetic retinopathy (DR)" and "Convolutional Neural Networks (CNN)"—in the abstract's first sentence. (Page 1)

Comment 2. In the abstract add more information about the results.
Reply: We expanded the abstract to include specific performance metrics, including accuracy, F1-scores, and Kappa on APTOS2019, Messidor-2, and EyePACS datasets. (Page 1)

Comment 3. Formatting of the introduction is not correct. Starting is of single column and rest is of two column.
Reply: The introduction has been carefully reformatted to maintain a consistent single-column layout throughout, in accordance with the official PLOS Computational Biology template (Version 3.6). Link: https://journals.plos.org/ploscompbiol/s/latex

Comment 4. The introduction should provide a stronger motivation for the study by clearly stating the research gap it addresses.
Reply: We revised the introduction to emphasize existing challenges in multiclass DR classification, including overreliance on preprocessing, limited explainability, and lack of uncertainty quantification.
To overcome this issue, a novel approach has been proposed in this work that directly utilizes fundus images with only basic resizing and normalization. It is important to distinguish this minimal preprocessing from the common practice of online data augmentation (e.g., random rotations and flips), which we employ during training to improve model generalization. To validate the robustness of our primary, preprocessing-free pipeline, we conduct a comparative analysis against versions of our model that incorporate intensive preprocessing steps, such as CLAHE

and Ben Graham's method. Our results confirm that the applied additional steps are unnecessary to achieve state-of-the-art performance with our proposed framework. (Page 3).

Comment 5. Format the paper correctly
Reply: We have carefully reviewed and standardized the formatting throughout the manuscript to ensure full compliance with the official PLOS Computational Biology template (Version 3.6). Link: https://journals.plos.org/ploscompbiol/s/latex

Comment 6. The objectives of the research should be explicitly outlined at the end of the introduction.
Reply: We thank the reviewer for this suggestion. The research objectives are stated at the end of the Introduction as: (Page 3)
This study aims to achieve robust and accurate multiclass DR classification and demonstrates the following contributions:

- Introduces a novel framework that integrates GCN with FE for multiclass DR classification using fundus images without excessive preprocessing.
- Employs a unique loss formulation that combines QA and UE losses with classification loss, enhancing model reliability and performance. QA evaluates the reliability of predictions using a fully connected layer trained with binary cross-entropy loss, and UE quantifies prediction confidence by calculating the variance across multiple stochastic passes through the network.
- Proposes a strategy that preserves image authenticity, enhancing robustness to real-world variations.
- Utilizes Grad-CAM heatmaps to strengthen interpretability and facilitate clinical validation and the significance of different input regions on the model's predictions.

Comment 7. At the end of the introduction add the organization of the paper.
Reply: A paragraph detailing the organization of the paper has been added at the end of the introduction section.
The rest of the paper is structured into distinct sections as follows: Section 2 reviews related works; Section 3 describes the methodology of the proposed graph convolutional networks-based diabetic retinopathy detection system; Section 4 shows the experimental setup, evaluation metrics; Section 5 presents the test results, compares the performance of our proposed model against established benchmarks, and includes an ablation study; Section 6 provides a discussion, and Section 7 concludes the study. (Page 3)

Comment 8. In fig 1, add the details for the CNN layer.
Reply: In the revised manuscript, Fig. 1 has been updated to include detailed information about the CNN layer architecture. (Page 2)

Comment 9. Caption of fig 2, is very long. Try to reduce it.
Reply: We shortened the caption and shifted technical explanations to the main text for better clarity and readability.
Model Architecture: The dataset $\mathcal{D}$ undergoes basic preprocessing (e.g., resizing, transformations, rotations) to prepare the data. The FE function f processes each sample $x \in \mathcal{D}$ to generate a feature vector (FV) $z \in \mathbb{R}^d$. This FV is then refined to a vector in $\mathbb{R}^d$ using Global Average Pooling (GAP). A graph is constructed with nodes corresponding to FVs, and the edge distance is computed based on spatial distance d_{sp} and semantic distance d_{se}. (Page 6)

Comment 10. In table 2, how the hyper-parameter values are selected.
Reply: We appreciate the reviewer's observation. All hyperparameters listed in Table 2 were chosen through empirical tuning on a validation set to ensure performance consistency and reproducibility.
It is worth mentioning that various hyperparameters of the applied models are automatically tuned employing the Optuna framework. (Page 10)

Comment 11. In table 4, add the year of publication.
Reply: We updated Table 4 entries to include the publication year alongside each reference. (Page 13)

Comment 12. Author can read the following papers to increase the technical strength of the paper.
Reply: We reviewed and cited the suggested papers. We also incorporated a comparison in the related works section, emphasizing how our method differs in terms of preprocessing, uncertainty modeling, and graph-based refinement.
Arora et al. [27] introduced an innovative deep learning framework leveraging EfficientNetB0 and CNN layering for accurate diabetic retinopathy diagnosis. Their model, trained on 35,108 retinal images, achieved an impressive 86.53% average accuracy and a 0.5663 loss rate. This robust computational approach offers precise and dependable classification of DR severity levels. Yadav et al. [28] developed a framework combining Modified Inertia Weight Particle Swarm Optimization (MIWPSO) and Fuzzy C-Means (FCM) for diabetic retinopathy image segmentation. This method achieved a remarkable 98.42% accuracy, significantly enhancing diagnostic capabilities by effectively eliminating noise and precisely segmenting medical images. (Page 4)

#Reviewer 2

Comment 1. The authors in "Related works" section critically refer to previous works like Feng et. al. as they test on limited dataset and not in realistic settings, raising this as one of the significant shortcomings. Nonetheless, the authors themselves test their model with only two standard datasets (APTOS2019 and Messidor-2). An external verification would help overcome the limitations highlighted in existing state-of-the-art methods.
Reply: We thank the reviewer for this insightful comment. In the revised manuscript, we have added Table 5 in the Results (Section 5), which reports the external validation results on the EyePACS dataset. These results demonstrate the model's robustness and generalizability to

real-world clinical data. (Page 14)
Table 5 presents the external validation performance on the EyePACS dataset using two top-performing backbones—DenseNet-169 (pretrained on Messidor-2) and MobilϵViT (pretrained on APTOS2019). After fine-tuning on EyePACS, both models demonstrated consistently high performance across all evaluation metrics: Accuracy, F1-score, AUROC, AUPR, and Cohen's κ. The applied DenseNet-169 technique achieved the best performance, with 97.38% accuracy and 99.83% AUROC. The demonstrated results suggest that the proposed framework is robust and transferable across datasets, addressing the common concern of limited dataset dependency in previous studies. (Page 12)

Table 1: **External validation on EyePACS:** Performance of the two best backbones from our pipeline—DenseNet-169 (initially optimized on Messidor-2) and MobilϵViT (initially optimized on APTOS2019)—after a brief fine-tuning stage on the EyePACS dataset.

**Initially Trained on**	**Backbone**	**EyePACS**				
		**Acc(%)**	**F1(%)**	**AUROC(%)**	**AUPR(%)**	**Kappa(%)**
Messidor-2	DenseNet169	97.38	97.37	99.83	99.39	96.72
APTOS2019	MobileViT	96.02	96.02	99.60	98.69	95.03

Comment 2. The role of data augmentation procedures (CLAHE, Ben Graham and rotations, flips, brightness adjustments) is not clear in the results. The authors mention as an advantage of their model (in the Introduction) not carrying out "extensive preprocessing," but they in fact make significant use of such preprocessing operations. It is undefined whether they use such procedures for comparison or as central constituents of their pipeline, defying their claim of "minimal preprocessing.
Reply: We clarified that our primary model uses minimal preprocessing (resize and normalization). CLAHE and Ben-Graham filtering were only used in comparative experiments, while standard augmentations (rotations, flips, brightness) were employed solely for training to improve class balance.
Following the reviewer's suggestion, we revised the manuscript as, To overcome this, we introduce a pioneering approach that directly utilizes real fundus images with only basic resizing and normalization. It is important to distinguish this minimal preprocessing from the common practice of online data augmentation (e.g., random rotations and flips), which we employ during training to improve model generalization. To validate the robustness of our primary, preprocessing-free pipeline, we conduct a comparative analysis against versions of our model that incorporate intensive preprocessing steps, such as CLAHE and Ben Graham's method. Our results confirm that these additional steps are unnecessary to achieve state-of-the-art performance with our proposed framework. (page 3)
Dataset Preparation: We first addressed the class imbalance problem by balancing the number of samples across the five DR classes to prepare our dataset for model training. We employed an oversampling approach for minority classes, ensuring that all five DR classes had an equal number of images for training. This balancing was achieved through a combination of duplicating samples using some transformations. These transformations are distinct from the intensive, dataset-wide preprocessing techniques that our primary model seeks to avoid. By following this procedure, the final class distribution across these batches was uniform, thereby mitigating bias in the model towards classes with higher initial representation. (Page 7-8)
Comparative Analysis of Preprocessing Techniques: A central claim of our work is that

our proposed GCN-enhanced framework performs robustly without requiring extensive image preprocessing. To support this claim, we designed experiments to compare our primary pipeline against two widely used preprocessing methods for DR classification. These methods were applied to the entire dataset before the training process and were evaluated separately from our main model.

(a) CLAHE: Contrast Limited Adaptive Histogram Equalization (CLAHE) is a contrast enhancement algorithm used to improve image contrast while preventing over-amplification. It enhances local contrast and is particularly effective in highlighting features in homogeneous regions [25].

(b) Ben-Graham Method: Ben Graham, a researcher in the deep learning domain, devised a preprocessing technique often used in medical image analysis tasks to improve images with varying lighting conditions, noise, or imbalance in contrast. This algorithm is used to enhance the features of the retinal fundus images and make the dataset more uniform by handling the variations in the brightness of the images [26].

The results of this comparative analysis, presented in Fig. 3, Fig. 4, and Fig. 5 and Table 4, evaluate the performance of our model under three conditions: (1) our proposed pipeline with no advanced preprocessing, (2) with CLAHE preprocessing, and (3) with Ben Graham preprocessing. (Page 11-12)

Comment 3. Explainability: a very brief explainability analysis is provided. The authors should clearly analyze how such regions change in alignment with severity outcome of DR as well as clearly mention their clinically relevant implication. It is stated in far too little detail.

Reply: We expanded our explanation of Grad-CAM results to highlight clinical relevance across DR severity levels. We also added detailed descriptions in the Results (Section 5) to demonstrate correlation with key retinal pathologies.

Explainable AI Visualization: To ensure the model's predictions are not only accurate but also interpretable, Grad-CAM is employed to visualize the regions influencing its classification decisions, as shown in Fig. 5. A detailed analysis of these heatmaps reveals that the model has learned to identify clinically relevant pathologies and that its focus correctly shifts in alignment with the increasing severity of Diabetic Retinopathy.

- **No DR (Class 0):** For fundus images of healthy retinas, the Grad-CAM activations are diffuse and lack a specific focus. This indicates the model is confirming the absence of key pathological markers, which is the desired behavior for a negative diagnosis.

- **Mild NPDR (Class 1):** In this early stage, the model's attention is drawn to small, punctate areas of high activation. These highlighted spots indicate the emergence of microaneurysms, which are the earliest signs of DR.

- **Moderate NPDR (Class 2):** As the disease progresses to the moderate stage, the activated regions on the heatmaps become larger and more pronounced. This shift in focus aligns with the clinical presentation of dot and blot hemorrhages and hard exudates, which are more significant vascular lesions than microaneurysms.

- **Severe NPDR & Proliferative DR (PDR) (Class 3 and 4):** In the most advanced stages, the Grad-CAM visualizations show large, intense areas of activation. These regions often correspond to significant retinal hemorrhages and, crucially, areas of neovascularization (the growth of new, abnormal blood vessels). The model's focus on these features, which are the indications of severe and proliferative DR, demonstrates its ability to recognize the most critical, vision-threatening signs of the disease.

This stage-by-stage analysis confirms that the framework bases its decisions on recognized clinical indicators of DR. The progressive shift in the model's attention from minor to major pathologies provides strong evidence of its clinical relevance and enhances trust in its utility as a reliable diagnostic tool. (Page 12 & 14-15)

Comment 4. There is not enough detail in uncertainty estimation description to reproduce. There is a need for clear description of implementation with exact parameters and seed selection settings.
Reply: We thank the reviewer for the constructive feedback. In response, we have revised the manuscript to provide a clearer and more detailed description of our uncertainty estimation procedure.
First of all, we revised the Uncertainty Estimation details under the Methodology (Section 3) to include a more complete description of the procedure.
In all experiments, we set the number of Monte Carlo (MC) forward passes to $T = 10$. Each pass keeps dropout active ($p = 0.3$ for the classifier head and $p = 0.2$ within the GCN layers), ensuring that different subnetworks are sampled on each pass. The final class-probability vector is computed as the arithmetic mean:

$$\bar{y} = \frac{1}{T}\sum_{t=1}^{T} \hat{y}^{(t)},$$

and the predictive uncertainty is measured as the standard deviation:

$$\sigma = \sqrt{\frac{1}{T}\sum_{t=1}^{T} \left(\hat{y}^{(t)} - \bar{y}\right)^2}.$$

To ensure reproducibility, we fixed all random seeds using: `torch.manual_seed(42)`, `torch.cuda.manual_seed_all(42)`, `np.random.seed(42)`. (Page 6-7)
Secondly, we reiterated the parameters used in the Uncertainty Estimation procedure under the implementation details of the Experiment section (Section 4).
During evaluation, the model performs $T = 10$ Monte Carlo dropout passes with dropout rates of $p = 0.3$ in the classifier and $p = 0.2$ in the GCN. Random seeds were fixed to 42 for PyTorch, CUDA, and NumPy to ensure exact reproducibility. (Page 10)

Lastly, we updated Table 4 so that it contains the Uncertainty Estimation hyperparameter values.

Table 2: Hyperparameter values used in our experiments

Category	Hyperparameter	Value	Description
Training Configuration	Number of Epochs	50	Total number of training epochs
	Batch Size	32	Samples per batch during training
	Learning Rate	5e-5	Initial learning rate
	Optimizer	AdamW	Optimization algorithm
	Weight Decay	0.01	L2 regularization parameter)
	Learning Rate Scheduler	ReduceLROnPlateau	Adjusts learning rate based on validation loss
	Scheduler Patience	7	Epochs with no improvement before reducing LR
	Early Stopping Patience	15	Epochs with no improvement before stopping training
Graph Construction	Number of Neighbors (k)	4	Nearest neighbors in graph construction
	Radius	0.1	Threshold for edge creation
	Feature Weight	0.5	Balance between spatial and semantic features
Uncertainty Estimation	MC Dropout Samples	10	Number of forward passes for Monte Carlo dropout
	Inference Dropout Rate	0.3	Dropout rate used during uncertainty estimation
GCN Configuration	Input Dimension	1024	Dimension of input features
	Hidden Dimensions	[512, 256]	Sizes of hidden layers
	Output Dimension	1024	Dimension of GCN output features
	Dropout Rate	0.2	Dropout applied to prevent overfitting
Loss Function	Classification Loss Weight	1.0	Weight for classification loss
	Quality Assessment Loss Weight	0.1	Weight for quality assessment loss
