# Supplementary material for: Graph-enhanced deep learning for diabetic retinopathy diagnosis: A quality-aware and uncertainty-driven approach
Source: PLoS Comput Biol. 2025 Dec 5;21(12):e1013745. doi: 10.1371/journal.pcbi.1013745 (PMC12680365; doi:10.1371/journal.pcbi.1013745)
Supplement: Attachment — Submitted filename: COMPBIOL-D-25-00649R1_Response.pdf [file pcbi.1013745.s002.pdf]

Manuscript Number: PCOMPBIOL-D-25-00649R1

Reply to Reviewers

Graph-Enhanced Deep Learning for Diabetic Retinopathy Diagnosis: A Quality-Aware and Uncertainty-Driven Approach

We would like to thank the reviewers for their comments. In the following, we will present our responses to the comments together with a summary of the corresponding changes in the revised manuscript in Yellow Highlights. All page numbers mentioned below are referred to in the new revised manuscript.

Reviewer 1

General Comment: The author addressed all the previous comments but still some issues are pending:

Comment 1. Check my previous comments. Some comments are not addressed correctly.
Reply: We carefully revisited the earlier comments and, to the best of our understanding, have now addressed all of them.

Comment 2. The abstract needs to be rewritten to point out significance and impact of the paper.
Reply: Following the reviewer's suggestion, we have rewritten the Abstract to discuss the paper's significance and impact: (i) the clinical burden and working-class implications of DR and the need for early detection, (ii) the contribution to trustworthy deployment via quality assessment and uncertainty estimation, and (iii) the performance and external generalization across APTOS2019, Messidor-2, and EyePACS, supported by Grad-CAM-based interpretability. (Page 1)

Comment 3. In the related work, it is recommended to refer the contribution made by the researchers and the novelty of the research.
Reply: We revised the Related Work to explicitly state each cited study's contribution and to clearly articulate our novelty relative to them; we added a synthesis paragraph highlighting common gaps (preprocessing, multiclass difficulty, single-dataset reliance, limited XAI) and included Table 1: Related work on Diabetic Retinopathy (DR): contributions, datasets, and gaps summarizing contributions and gaps to make the contrast explicit. (Page 6)

Comment 4. I recommend that the authors add some more current articles to improve the paper's overall quality. The preparation of a comparative analysis of the current publications on this subject should also be included.
Reply: We have added recent articles of 2025 in the Related works section and Comparison Table (Table 5: Comparison with SOTA), [18], [24] and [25]. (Page 5 and Page 14)

Comment 5. Avoid presenting with lengthy paragraph
Reply: We have carefully reviewed and avoided lengthy paragraphs.

Comment 6. The references section lacks key foundational or recent works that are crucial to the topic.
Reply: We have added recent articles of 2024 and 2025 in the References section, e.g., [2], [5], [12], [14], [18], [19], [20], [21], [23], [25], etc. (Page 19-22)

Comment 7. Ensure that all cited works are relevant and up-to-date to reflect the current state of research.
Reply: Thanks. Checked.

Comment 8. A more balanced mix of theoretical and empirical studies in the citations would strengthen the paper.
Reply: Thank you for the suggestion. We revised the Related Work to include a more balanced mix of theoretical foundations (graph learning, calibration/uncertainty, interpretability) and empirical DR studies/benchmarks (APTOS2019, Messidor-2, EyePACS), and we updated the synthesis and Table 1: Related work on Diabetic Retinopathy (DR): contributions, datasets, and gaps accordingly. (Page 4–6)

Comment 9. Finally, a final proof-reading is highly suggested, in order to correct some typos.
Reply: Following what the reviewer suggested, we have proofread the revised manuscript by a Technical Writing Specialist, North South University, Bangladesh. Additionally, we have checked the entire revised paper with a premium subscription account of Grammarly, which is a cloud-based typing assistant that reviews spelling, grammar, punctuation, clarity, engagement, and delivery mistakes.

#Reviewer 2

General Comment: I find that the authors have responded well to my concerns. They added an external validation set, which strengthens the consistency and generalizability of their method; indeed the method still performs well. They also better explained the preprocess problem making their statements more clear/robust On the explainability section, they expanded the description and linked it to the clinical interpretability. Regarding the uncertainty estimation part, they explained the environment for their implementation, making it possible to reproduce their approach.

I think now the article is clearer, more complete, and methodologically adequate
Reply: Thank you for your thoughtful and encouraging assessment; we're grateful that the added external validation, clearer preprocessing rationale, expanded explainability, and reproducibility details strengthened the manuscript.

Comment 1. I recommend adding a README file to the GitHub repository to enhance the

reproducibility of the work. I would include instructions on installing dependencies, preparing the dataset, running the code, and reproducing the main result.
Reply: We have added a comprehensive README to the repository covering dependency installation (requirements/conda), dataset preparation, run commands, and step-by-step instructions (with seeds/configs) to reproduce the main results
